# Cytotoxic and Antibacterial Activity of Koninginins Isolated from the Mangrove-Derived Endophytic Fungus *Trichoderma* sp.

**DOI:** 10.3390/molecules29225278

**Published:** 2024-11-08

**Authors:** Gisele da Costa Ramos, Ingryd Nayara de Farias Ramos, Luciano Almeida Watanabe, Luciana Almeida Watanabe Castro, Alessandra Jackeline Guedes de Moraes, Gleiciane Rodrigues dos Santos, José Edson de Sousa Siqueira, André Salim Khayat, Andrey Moacir do Rosario Marinho, Patrícia Santana Barbosa Marinho

**Affiliations:** 1 Chemistry Graduate Program, Federal University of Pará, Rua Augusto Corrêa, 01, Belém 66075110, Brazil; giamajesus@gmail.com (G.d.C.R.); lucianowat@yahoo.com.br (L.A.W.); siqueira.edson@outlook.com (J.E.d.S.S.); andrey@ufpa.br (A.M.d.R.M.); 2 Oncology Research Center, Federal University of Pará, Rua dos Mundurucus, 4487, Belém 66073005, Brazil; ingrydramos7@gmail.com (I.N.d.F.R.); khayatas@gmail.com (A.S.K.); 3 Evolution Laboratory, Federal University of Pará, Alameda Leandro Ribeiro, Bragança 68600000, Brazil; lwatanabe@ufpa.br; 4 Institute of Agricultural Sciences, Federal Rural University of the Amazon, Avenida Presidente Tancredo Neves, 2501, Belém 66077830, Brazil; alessandra.moraes@uepa.br (A.J.G.d.M.); gleicianerodrigues8@gmail.com (G.R.d.S.)

**Keywords:** koninginins, cytotoxic activity, *thichoderma*, amazon

## Abstract

The search for bioactive compounds for the treatment of several diseases has led to the study of endophytic fungi. Neoplastic diseases are among the most significant health concerns due to their high mortality rate, and there is a dearth of efficacious pharmaceutical agents for the treatment of cancer. Gastric cancer is one of the most aggressive forms of cancer and is among those with the highest mortality rates in Brazil. Accordingly, the objective of this study was to identify compounds with cytotoxic activity from the mangrove-derived endophytic fungus *Trichoderma* sp. Isolation of the chemical compounds was conducted using chromatographic methods, while structural elucidation was achieved through the application of spectroscopic (NMR and UV) and spectrometric (MS) techniques. The fungus *Trichoderma* sp. was found to produce five distinct koninginins (A, B, C, E, and J). The organic phases of the extracts and isolated compounds were evaluated for their antimicrobial and cytotoxic potentials, respectively, through microdilution testing and the MTT method. In the cytotoxicity assay, both the AF extract and koninginin A demonstrated favorable outcomes, indicating their potential as promising anticancer therapeutic agents.

## 1. Introduction

Cancer is a general term that encompasses a range of diseases with the potential to affect any part of the body. Related terms include malignant tumors and neoplasms. A defining characteristic of cancer is the formation of aberrant cells that proliferate uncontrollably, invading nearby tissues and spreading to other organs. This process, known as metastasis, is the leading cause of cancer-related mortality [1].

The rising incidence of cancer represents a significant challenge to the treatment of this disease, given its complex nature and the intricacies of its treatment. Cancer is a progressive process that involves the interaction of genetic and environmental factors and the dysfunction of various cell mechanisms, including those responsible for DNA repair, apoptosis, and immunological functions [2].

As estimated by GLOBOCAN 2020, the global incidence and mortality of cancer in 2020 reached 19.3 million new cases and 10.0 million deaths, respectively [3]. In Brazil, it is projected that 704,000 new cases of cancer will emerge over the three-year period spanning 2023 to 2025 [4]. The most prevalent forms of cancer include gastric cancer and melanoma. Approximately 780,000 deaths per year are attributed to gastric cancer, which represents the fourth most lethal form of cancer globally and accounts for 8.3% of all cancer-related mortalities [5]. Gastric cancer is one of the most prevalent forms of cancer in Brazil. Adenocarcinoma represents 95% of cases, with the majority of patients being male individuals between the ages of 60 and 70 [6].

The already well-documented phenomenon of bacterial resistance represents a significant challenge to global public health. Bacterial infections can range in severity from a relatively minor inflammation, such as a sore throat, to a potentially fatal condition. The search for new compounds with cytotoxic and antimicrobial activity has intensified, as has the search for more effective and selective treatments or new strategies to prevent the progression of these diseases [7,8]. A number of compounds documented in the scientific literature have demonstrated activity in both prokaryotic cells (bacteria) and eukaryotic cells (cancer cells) [9,10]. While this may be a beneficial property, the broad spectrum of action may also indicate a lack of selectivity for a specific target. In addition, compounds that do not exhibit pronounced antimicrobial activity may be specific for eukaryotic targets. Consequently, we also assess antimicrobial activity in our research to confirm the efficacy of our compounds and to determine whether they demonstrate selectivity.

It is, therefore, imperative that the pharmaceutical industry identify and develop new compounds to address the growing prevalence of antibiotic-resistant bacteria and provide more effective alternatives for cancer treatment. Endophytic fungi are significant sources of bioactive compounds with biological activity, as evidenced by previous research [11,12]. Thus, in this study, we report the isolation, cytotoxic, and antimicrobial activity of koninginin compounds from the mangrove-derived endophytic fungus *Trichoderma* sp.

## 2. Results

### 2.1. HPLC-DAD of AF Extract

**Figure 1:**
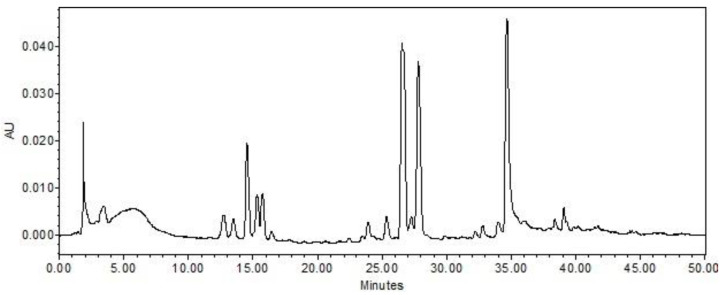
Chromatogram of the AF extract of the fungus *Trichoderma* sp. AcCC18.2 in wavelength at 254 nm. Chromatogram obtained at 254 nm on an Alliance e2695 line chromatograph with photodiode-array detector and Sunfire C18 reversed-phase column. Gradient H_2_O/MeOH 95:5 to 0:100 in 60 min; flow of 1.0 mL/min; column oven temperature 40 °C; injection volume 20 μL; concentration of 1 mg/mL.

As illustrated in Figure 1, the AF extract chromatogram exhibits ten predominant peaks. The ultraviolet–visible (UV–Vis) spectra of the aforementioned peaks exhibit an absorption maximum at wavelengths between 260 and 270 nanometers, which is consistent with the characteristics of koninginin polyketides, a class of secondary metabolites commonly produced by fungi of the *Trichoderma* genus [13,14,15,16,17].

### 2.2. Isolation and Identification of the Koninginins

**Figure 2:**
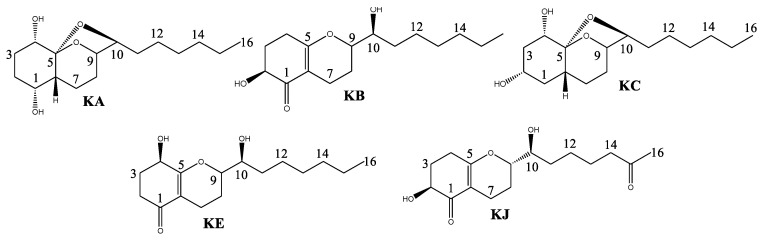
Compounds isolated from extract AF of the *Trichoderma* sp.: Koninginin A (KA), Koninginin B (KB), Koninginin C (KC), Koninginin E (KE), and Koninginin J (KJ).

The fractionation of the AF extract by CC resulted in the isolation of koninginin compounds. Secondary metabolites of this class are polyketides found in fungi of the genus *Trichoderma*. They are structurally characterized as an analog of the octahydro-2*H*-1-benzopyran cycle with a seven-carbon side chain directly linked at C-3 [16,18,19]. All koniginins isolated in this study exhibited oxidation at C-10 in the side chain, as confirmed by ^13^C NMR signals between 73.0 and 79.5 for the compounds. Koniginins KA and KC possess an additional five-membered ring, which forms an acetal group. This is evidenced by the presence of ^13^C NMR signals at 109.2 (KA) and 108.5 (KC). Koningins KB, KE, and KJ lack the acetal ring, exhibiting instead a C=C double bond, as evidenced by the presence of signals at C-5 (~δ 170.0) in ^13^C NMR. Furthermore, the Koningin compounds KA and KC display two hydroxylated carbons in ring A, which are situated at C1, C2, and/or C4, as indicated by their HSQC and HMBC correlations. In contrast, Koningins KB, KE, and KJ display the expected signals for the carbonyl group at C-1, as revealed by their HMBC correlations. Accordingly, the Koningins (Figure 2) were identified as Koninginin A (KA), Koninginin B (KB), Koninginin C (KC), Koninginin E (KE), and Koninginin J (KJ) [14,16,18].

### 2.3. Antimicrobial Assays

The antimicrobial activity of the extracts (HF, AF, and HEF) and isolated koninginins was evaluated against both Gram-positive and Gram-negative bacteria. The results of the antimicrobial tests demonstrate that only the AF extract and the substances KA and KE exhibit some degree of bacterial activity (Table 1). Amoxicillin was employed as a positive control.

### 2.4. Cytotoxic Activity

The objective of the single-dose experiment was to identify the substances with the most effective inhibition percentages through the utilization of the MTT assay. The results demonstrated that the AF extract exhibited the most pronounced inhibitory effect, with a percentage inhibition exceeding 50% in all tumor cell models tested. The AF extract demonstrated superior efficacy compared to the positive control (5-FU). The results are presented in tabular form in Table 2.

Following an analysis of the single dose results, the AF extract was selected for a dose–response curve experiment with the objective of obtaining IC_50_ values in the five tumor cell lines as well as in the non-tumor cell line. Therefore, the AF extract demonstrated cytotoxic potential in all tested lineages, with IC_50_ values below 50 μg/mL. This potential was most pronounced on the metastatic melanoma cell line (SK-MEL 19), with an IC_50_ of 22.18 μg/mL, as illustrated in Table 3.

**Figure 3:**
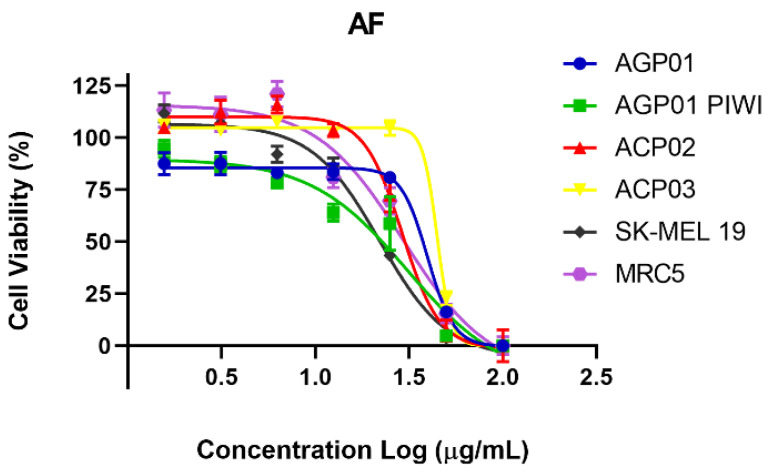
Cell viability percentage curve after 72 h of treatment with AF extract in different cell lines *. Each point equals the average of three replicates. * AGP-01 (intestinal-type gastric adenocarcinoma), AGP-01 PIWIL1^-/-^ (intestinal-type gastric adenocarcinoma with inactivated *PIWIL1* gene)1, ACP02 (diffuse-type gastric adenocarcinoma) and ACP03 (diffuse-type gastric adenocarcinoma), SK-MEL19 (human metastatic melanoma), and MRC5 (normal human fibroblasts).

The MTT assay was also employed to indirectly assess the impact of the AF extract on cell viability. The MTT assay was also utilized to indirectly evaluate the influence of the AF extract on cellular viability. The number of viable cells exhibited a concentration-dependent decline with increasing extract concentration, as observed across all cell lines tested (Figure 3).

### 2.5. Cytotoxic Activity of Koninginins Isolated from Trichoderma sp. AcCC18.2

To evaluate the activity of koninginins A, B, and E, which were isolated from the mangrove-derived endophytic fungus *Trichoderma* sp., a single-dose experiment was conducted in the aforementioned tumor models. The results are presented in Table 4.

Koninginin A demonstrated a notable inhibitory effect on the gastric cancer models employed in this study. To ascertain its average inhibitory concentration (IC_50_), a concentration-response curve was constructed. As evidenced in Table 5, koninginin A exhibits cytotoxic activity against both AGP01 and ACP02 cell lines, with IC_50_ values of 36.43 and 40.19 μg/mL, respectively.

**Figure 4:**
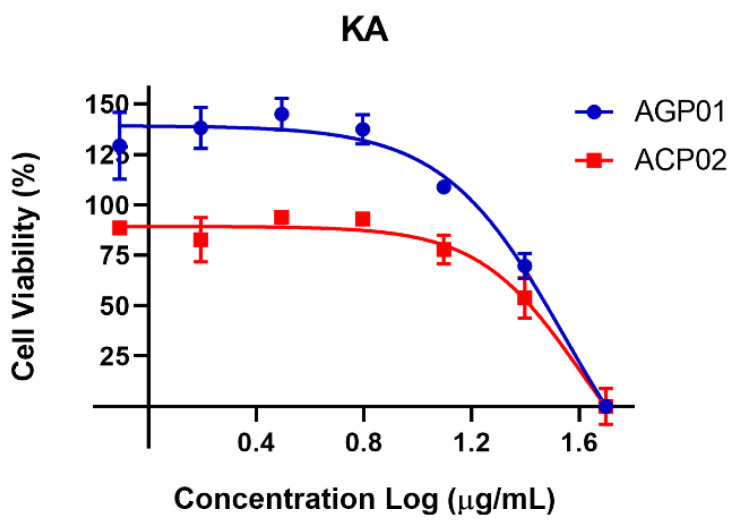
Cell viability percentage curve after 72 h of treatment with koninginin A (KA) in two gastric cancer cell lines *. Each point equals the average of three replicates. * AGP-01 (intestinal-type gastric adenocarcinoma) and ACP02 (diffuse-type gastric adenocarcinoma).

Furthermore, an inverse correlation was identified between cell viability and increasing koninginin A concentrations. To illustrate the concentration-dependent effect of this molecule, the data are presented in Figure 4.

## 3. Discussion

The search for secondary metabolites with cytotoxic activity has been widely documented in the context of the development of new agents for the treatment of cancer [20,21]. The isolation of compounds belonging to the class of koninginins from fungi of the genus *Trichoderma* has been reported in several scientific works [22,23,24]. Nevertheless, the cytotoxic potential of koninginins against cancer cells has been little studied. In this study, the cytotoxic potential of compounds KA, KB, and KE was evaluated against pathogenic bacteria and their ability to inhibit the growth of gastric cancer tumor cells, which is one of the most aggressive cancers with high incidence and mortality in Brazil. The compounds KC and KJ were not subjected to testing due to the insufficient quantities and purity of the samples obtained.

The extracts and koninginins KA, KB, and KE demonstrated minimal antimicrobial activity against both Gram-positive and Gram-negative bacteria, corroborating prior reports that these compounds exhibit comparable activities against the bacteria tested [14,16,25,26]. However, they demonstrated notable cytotoxic activity on the AGP-01 cell, which may indicate that these compounds are more active and exhibit greater selectivity of action for eukaryotic cells [27]. It is already established that natural products can inhibit the elongation phase of eukaryotic protein biosynthesis [28]. This selectivity may prove beneficial, as it could enhance the specificity of the compound, thereby improving the efficacy of an antitumor treatment.

Fungal extracts have demonstrated relevant cytotoxic activity, as evidenced by preliminary studies such as that conducted by Wu et al. (2014) [29], which evaluated the anticancer activities of various fungal species against diverse cancer cell lines. The ability of the extracts to inhibit tumor cell growth was observed in these studies. Moreover, acetone extracts of the endophytic fungus EL002332, isolated from *Endocarpon pusillum*, demonstrated selective cytotoxicity against AGS human gastric cancer cells [30]. In our cytotoxicity assays, the AF extract demonstrated good activity against the metastatic melanoma cell line (SK-MEL 19), with an IC_50_ value of 22.18 μg/mL. Furthermore, the IC_50_ was observed to be 39.91 μg/mL against the metastatic gastric cancer cell (AGP01).

While the activity of extracts is already a significant factor in the search for new drugs, it is nevertheless important to have a detailed understanding of their chemical composition, even at the level of major metabolites. This knowledge can be used to enhance the desired activity by isolating the bioactive compound. Therefore, among the extracts evaluated, the ethyl acetate phase (AF) was identified as the most promising and was subjected to fractionation for the isolation of compounds, suggesting that the koninginins present in the extract may be responsible for the observed activity. Consequently, koninginins A, B, and E were subjected to testing for their cytotoxic activities.

In the cytotoxicity test, it was observed that KA demonstrated an inhibition percentage exceeding 50% in the metastatic gastric cancer model (AGP01) and approaching 50% in the primary gastric cancer models (ACP02 and ACP03). This indicates that KA exhibited preferential activity in gastric cancer models. While koninginins B and E did not demonstrate a notable degree of inhibition in any of the cell lines examined, Koninginin A demonstrated 18 times the activity of KB and 10 times the activity of KE against AGP01, a lineage representing a model of metastatic gastric cancer. This suggests a potential selectivity in the cytotoxicity of more advanced cancer cells, which may be less susceptible to treatment than primary tumors. The discrepancy in activity observed between KA and KB and between KA and KE may be attributed to the presence of the C-O-C ether bridge that forms the acetal ring in KA, which is absent in KB and KE. Nevertheless, further investigation is required to elucidate the structure-activity relationship and the general mechanism of action. Nevertheless, our findings suggest that koninginins, particularly koninginin A, may represent a promising alternative strategy for combating gastric cancer cells. These findings open up new possibilities in the therapeutic arsenal against neoplastic diseases.

## 4. Materials and Methods

### 4.1. Microorganism

The strain of *Trichoderma* sp. utilized in this study was procured from the Laboratory of Bioassays and Chemistry of Microorganisms at the Federal University of Pará (UFPA), cataloged as AcCC18.2. The fungus was identified through the use of molecular biology techniques.

### 4.2. Cultivation of Fungus and Obtaining Extracts

The mangrove-derived endophytic fungus, *Trichoderma* sp. AcCC18.2, was initially reactivated in a Petri dish containing PDA culture medium (potato, dextrose, agar) and cultivated for seven days. Subsequently, small fragments of the fungus were inoculated into 10 sterile Erlenmeyer flasks (500 mL) containing 100 g of rice and 75 mL of distilled water in a laminar flow hood (PANCHANE^®^, model PA 320). The flasks were maintained in a static state at room temperature for 26 days to facilitate colony growth, with two flasks serving as controls. Subsequently, 400 mL of 92% ethanol was added to each bottle containing the biomass. Following a 72-h period, the material was filtered, and the solution obtained was evaporated in a rotary evaporator to reduce its volume. The concentrated ethanolic solution was subjected to liquid-liquid partition using hexane and ethyl acetate, resulting in the separation of three distinct phases: hexane, ethyl acetate, and hydroalcoholic. Following concentration via rotary evaporation, the resulting phases exhibited the following yields: hexane phase (HF) 6 g, ethyl acetate phase (AF) 24 g, and hydroethanolic phase (HEF) 15 g.

### 4.3. HPLC-DAD of Ethyl Acetate Phase Extract (AF)

The AF extract was subjected to high-performance liquid chromatography (HPLC) to obtain its chromatographic profile on an Alliance e2695 chromatograph (Waters^®^), which was equipped with an autosampler and a photodiode-array detector (DAD, Waters 2998). The ultraviolet–visible range spanned 210 to 600 nm. The separations were conducted on a Sunfire C18 reverse-phase column (150 mm × 4.6 mm internal diameter, 5 μm particle size; Waters, Wexford, Ireland) with a C18 precolumn (20 mm × 4.6 mm internal diameter, 5 μm particle size, Waters). The chromatography system was operated using Empower 3 Personal Single System software (Waters Corparation, Milford, MA, EUA). The linear exploratory gradient employed was H_2_O/ACN 95:5 to 0:100 over a 60-min period at a flow rate of 1.0 mL/min. The column temperature was maintained at 40 °C, and 20 μL of the sample was injected at a concentration of 1 mg/mL.

### 4.4. Fractionation of AF Extracts from the Fungus Trichoderma sp. AcCC18.2

A total of 5 g of AF extract was subjected to fractionation in a chromatographic column on a silica gel (CC) column (Silia Flash F60 particles of 230–400 mesh, Silicycle^®^). The mobile phase mixtures were composed of hexane, ethyl acetate (EtOAc), and methanol (MeOH) (Tedia^®^), with the polarity of the mixtures gradually increasing in a stepwise manner. The resulting fractions were as follows: hexane (Fr01), hexane/EtOAc 30% (Fr02), hexane/EtOAc 50% (Fr03), hexane/EtOAc 70% (Fr04), EtOAc (Fr05), EtOAc/MeOH 30% (Fr06), and MeOH (Fr07). The fractions were monitored by thin-layer chromatography (TLC). Following repeated CC purifications, compounds KB (66.8 mg) and KC (15.2 mg) were obtained from the Fr04 fraction, while compounds KA (8.1 mg), KE (4.0 mg), and KJ (3.4 mg) were isolated from fraction Fr05.

### 4.5. NMR and MS Analysis of the Isolated Compounds

Nuclear magnetic resonance (NMR) spectra were obtained using a Bruker Ascend 400 spectrometer (Bruker, Bremen, Germany). The samples were dissolved in CDCl_3_ and MeOD. TMS was employed as an internal standard for calibration of the spectra. The coupling constants (*J*) were measured in Hertz, and the chemical shifts were reported on a delta scale (δ). Mass spectra were obtained using an Acquity TQD H-class spectrometer (Waters, Mississauga, ON, Canada), operated in both negative and positive electrospray ionization modes.

### 4.6. Antimicrobial Assays

Antimicrobial assays were conducted using certified strains of *Bacillus subtilis* (ATCC 6633), *Escherichia coli* (ATCC 25922), *Staphylococcus aureus* (ATCC 25923), *Salmonella typhimurium* (ATCC 14028), and *Pseudomonas aeruginosa* (ATCC 27853), obtained from the Instituto Evandro Chagas in Brazil. The extracts and isolated compounds were evaluated by microdilution in 96-well plates to verify the minimum inhibitory concentration (MIC) in accordance with the recommendations of the Clinical and Laboratory Standards Institute [31]. The results were observed by adding 10 µL of TTC (2,3,5-triphenyltetrazolium chloride) in accordance with the methodology by Ramos et al. (2022) [32].

### 4.7. In Vitro MTT (3-(4,5-Dimethylazol-2-yl)-2,5-Dephenitetrazolium Bromide) Assay

The tumor cell lines utilized in this study were SK-MEL19 (human metastatic melanoma), AGP-01 (intestinal-type gastric adenocarcinoma), AGP-01 PIWIL1^-/-^(intestinal-type gastric adenocarcinoma with inactivated PIWIL1 gene), ACP02 (diffuse-type gastric adenocarcinoma), and ACP03 (diffuse-type gastric adenocarcinoma). The non-tumor lineage cell lines included MRC5 (human lung fibroblast). The cells were cultivated in adherent monolayer cultures in Dulbecco’s Modified Eagle’s Medium (DMEM, Gibco^®^), supplemented with 10% fetal bovine serum and penicillin (100 U/mL) and streptomycin (100 mg/mL) (Gibco^®^). The cultures were maintained in an incubator at 37 °C with a 5% CO₂ atmosphere. An initial single-dose experiment was conducted to identify the optimal molecules and cells. Subsequently, a dose–response curve experiment was conducted to determine the mean inhibitory concentration (IC_50_). For this purpose, cells were seeded in 96-well plates at a density of 3 × 10³ cells/well for a period of 24 h to allow for adhesion to the plate. Subsequently, the cells were treated with test samples at single dose concentrations, namely, extracts at 50 μg/mL and compounds at 10 μg/mL, with a curve of 1.56 to 100 μg/mL, and incubated at 37 °C for 72 h. The samples were dissolved in dimethyl sulfoxide (DMSO) to obtain the final concentration, and the experiments were performed in triplicate. The negative control was the untreated sample, while the positive control was the chemotherapeutic agent 5-Fluorouracil (5-FU) at a concentration of 10 μg/mL. Following the designated treatment period, 100 μL of MTT solution (stock solution 5 mg/mL, diluted 1:10 in DMEM medium) was added to each well of the plate and incubated at 37 °C for 3 h. The subsequent analysis was conducted using a plate spectrophotometer (SYNERGY/HT microplate reader) at a wavelength of 570 nm.

### 4.8. Data Analysis

The percentage of inhibition induced by the samples on the strains was calculated using Equation (1) in the Microsoft Excel version 10 program as part of the single-dose experiment.

ABS Treated × 100 ÷ ABS NC(1)

where ABS—Absorbance, and NC—Negative Control.

The mean inhibitory concentration (IC_50_) and its respective confidence intervals (CI_95%_) were determined using a sigmoidal dose–response equation (non-linear regression), as outlined in Equation (2) in GraphPad Prism, version 8.

Y = 100 ÷ 1 + 10 (LogIC_50_ − x) × HillSlope (2)

## 5. Conclusions

The initial cytotoxicity and cell viability tests revealed that the AF extract demonstrated cytotoxic activity in all neoplastic cell lines examined. The isolation of compounds from the AF extract demonstrated that koninginins, particularly koninginin A, possess cytotoxic activity against tumor cells and reduce cell viability. The antimicrobial activity of compounds KA, KB, and KE was found to be relatively weak. This may be attributed to a certain degree of selectivity exhibited by these compounds towards eukaryotic models and cells. This illustrates the significance of the isolation and characterization of natural compounds, as the elucidation of their activity can offer valuable insights into the field of cancer pharmacology.

## Figures and Tables

**Table 1:** Antimicrobial assays of extracts and compounds obtained from mangrove-derived endophytic fungus *Trichoderma* sp. AcCC18.

Sample	MIC (µg/mL)				
	Bs ^g^	Ec ^h^	Pa ^i^	St ^j^	Sa ^k^
**AF ^a^**	500 (=); 125 (−)	>500	>500	NT	NT
**HF ^b^**	>500	>500	>500	NT	NT
**HEF ^c^**	>500	>500	>500	NT	NT
**KA ^d^**	250 (=)	>500	>500	>500	500 (−)
**KB ^e^**	>500	>500	>500	>500	500 (−)
**KE ^f^**	500 (=); 250 (−)	>500	>500	>500	500 (=)
**Amoxicillin**	7.81 (=)	7.81 (=)	125 (=)	7.81 (=)	7.81 (=)

^a^ Ethyl acetate phase. ^b^ Hexanic phase. ^c^ Hydroethanolic phase. ^d^ Koninginin A. ^e^ Koninginin B. ^f^ Koninginin E. ^g^ *Bacillus subtilis.* ^h^ *Escherichia coli.* ^i^ *Pseudomonas aeruginosa*. ^j^ *Salmonella typhimurium*. ^k^ *Staphylococcus aureus*. Type of activity = Bactericide; − Bacteriostatic. NT = not tested. Positive control—Amoxicillin.

**Table 2:** Percentage values of cell growth inhibition after treatment at 50 µg/mL of extract in 72 h of incubation for five tumor cell lines (SK-MEL-19, AGP01, AGP-01 PIWI^-/-^, ACP02, and ACP03).

Inhibition Percentage (%)					
Extracts	Cell Lines				
	AGP01	AGP01 *PIWIL1*^-/-^	ACP02	ACP03	SK-MEL 19
**NC ^a^**	0	0	0	0	0
**5-FU ^b^**	47	50	40	46	48
**AF ^c^**	54	63	51	51	56
**HF ^d^**	13	20	16	16	−3
**HEF ^e^**	23	21	23	22	14

^a^ Negative control. ^b^ 5-Fluorouracil. ^c^ Ethyl acetate phase. ^d^ Hexanic phase. ^e^ Hydroethanolic phase.

**Table 3:** Cytotoxic activity of AF extract on cell lines after 72 h of exposure.

IC_50_ (μg/mL) *						
Extracts	Cell Lines					
	AGP01	AGP01 *PIWIL1*^-/-^	ACP02	ACP03	SK-MEL 19	MRC5
**AF ^a^**	39.91 (37.14–42.89) R^2^= 0.9894	31.37 (20.62–47.72) R^2^= 0.9358	28.97 (26.78–31.33) R^2^= 0.9858	45.34 (19.24–106.8) R^2^= 0.9963	22.18 (19.86–24.77) R^2^= 0.9849	29.17 (21.2–40.12) R^2^= 0.9591

^a^ Ethyl acetate phase. * Data are presented as IC_50_ values and 95% confidence intervals obtained by non-linear regression for all cell lines from three independent experiments.

**Table 4:** Percentage values of cell growth inhibition after treatment with isolated koninginins, at 40 µg/mL in 72 h of incubation, for five tumor cell lines.

Inhibition Percentage (%)					
Cell Lines					
Sample	AGP01	AGP01 *PIWIL1*^-/-^	ACP02	ACP03	SK-MEL 19
**NC ^a^**	0	0	0	0	0
**5-FU ^b^**	47	50	40	46	48
**KA**	54	42	46	45	36
**KB**	3	8	14	2	10
**KE**	5	3	−2	−4	7

^a^ Negative control. ^b^ 5-Fluorouracil.

**Table 5:** Cytotoxic activity of koninginin A on gastric cancer cell lines after 72 h of exposure.

IC_50_ (μg/mL) *		
Cell Lines		
Sample	AGP01	ACP02
**KA ^a^**	36.43 (24.36–39.52) R^2^ = 0.9752	40.19 (38.22–45.87) R^2^ = 0.9624

* Data are presented as IC_50_ values and 95% confidence intervals obtained by non-linear regression for all cell lines from three independent experiments. ^a^ Koninginin A.

## Data Availability

Data are contained within the article and Supplementary Materials.

## Supplementary Materials

The following supporting information can be downloaded at https://www.mdpi.com/article/10.3390/molecules29225278/s1: Figure S1: ^1^H NMR spectrum (400 MHz, CDCl_3_) of Koninginin A (KA). Figure S2: ^13^C NMR spectrum (100 MHz, CDCl_3_) of Koninginin A (KA). Figure S3: ^1^H NMR spectrum (400 MHz, CDCl_3_) of Koninginin B (KB). Figure S4: ^13^C NMR spectrum (100 MHz, CDCl_3_) of Koninginin B (KB). Figure S5: ^1^H NMR spectrum (400 MHz, CDCl_3_) of Koninginin A (KA) and C (KC). Figure S6: ^13^C NMR spectrum (100 MHz, CDCl_3_) of Koninginin A (KA) and C (KC). Figure S7: ^1^H NMR spectrum (400 MHz, CDCl_3_) of Koninginin E (KE). Figure S8: ^13^C NMR spectrum (100 MHz, CDCl_3_) of Koninginin E (KE). Figure S9: ^1^H NMR spectrum (400 MHz, CDCl_3_) of Koninginin J (KJ). Figure S10: ^13^C NMR spectrum (100 MHz, CDCl_3_) of Koninginin J (KJ). Figure S11: Cell viability percentage curve after 72 h of treatment with AF extract in different cell lines*. Each point equals the average of three replicates. Figure S12: Cell viability percentage curve after 72 h of treatment with Koninginin A (KA) in two gastric cancer cell lines*. Each point equals the average of three replicates.
