# Supplementary material for: Cytotoxic and Antibacterial Activity of Koninginins Isolated from the Mangrove-Derived Endophytic Fungus Trichoderma sp
Source: Molecules. 2024 Nov 8;29(22):5278. doi: 10.3390/molecules29225278 (PMC11596921; doi:10.3390/molecules29225278)
Supplement: Supplementary file 1 [file molecules-29-05278-s001.zip › molecules-3255467-supplementary.pdf]

molecules

Supplementary Materials

Table of Contents

Figure S1: ^{1}H NMR spectrum (400 MHz, $CDCl_3$) of Koninginin A (KA)

Figure S2: ^{13}C NMR spectrum (100 MHz, $CDCl_3$) of Koninginin A (KA)

Figure S3: ^{1}H NMR spectrum (400 MHz, $CDCl_3$) of Koninginin B (KB)

Figure S4: ^{13}C NMR spectrum (100 MHz, $CDCl_3$) of Koninginin B (KB)

Figure S5: ^{1}H NMR spectrum (400 MHz, $CDCl_3$) of Koninginin C (KC)

Figure S6: ^{13}C NMR spectrum (100 MHz, $CDCl_3$) of Koninginin C (KC)

Figure S7: ^{1}H NMR spectrum (400 MHz, $CDCl_3$) of Koninginin E (KE)

Figure S8: ^{13}C NMR spectrum (100 MHz, $CDCl_3$) of Koninginin E (KE)

Figure S9: ^{1}H NMR spectrum (400 MHz, $CDCl_3$) of Koninginin J (KJ)

Figure S10: ^{13}C NMR spectrum (100 MHz, $CDCl_3$) of Koninginin J (KJ)

Figure S11: Cell viability percentage curve after 72 hours of treatment with AF extract in different cell lines*. Each point equals the average of three replicates.

Figure S12: Cell viability percentage curve after 72 hours of treatment with koninginin A (KA) in two gastric cancer cell lines*. Each point equals the average of three replicates.

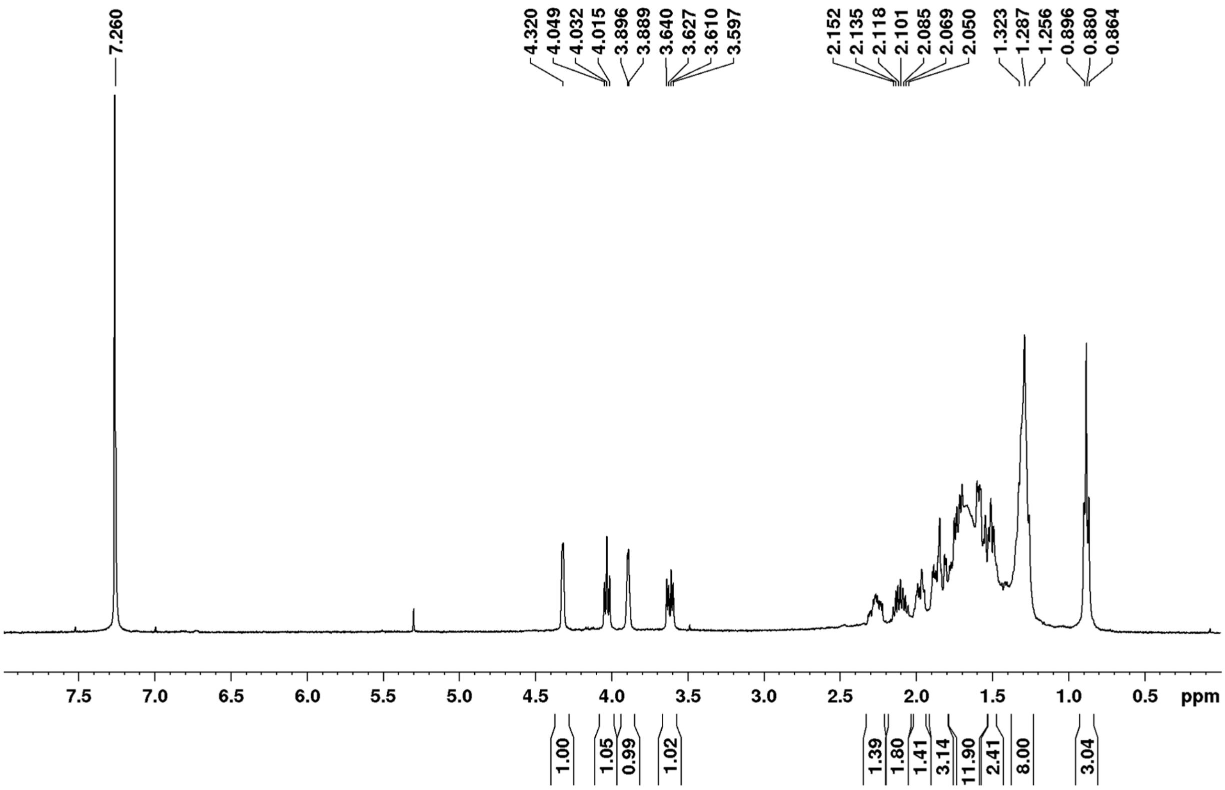

Figure S1: ^{1}H NMR spectrum (400 MHz, $CDCl_3$) of Koninginin A (KA)

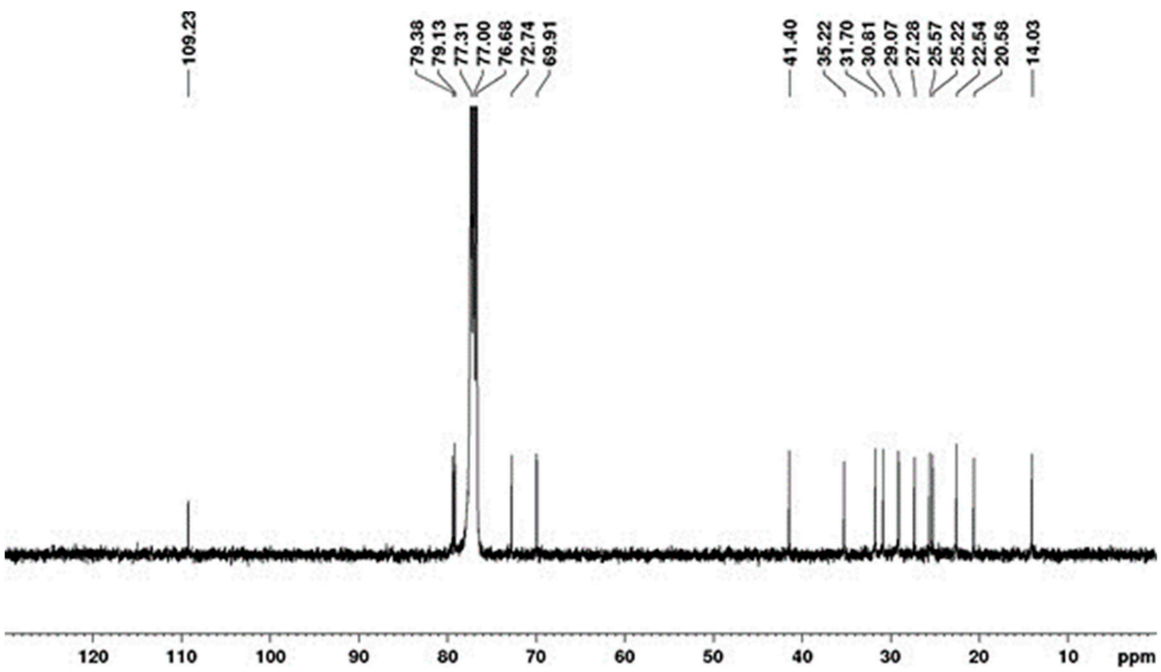

Figure S2: ^{13}C NMR spectrum (100 MHz, $CDCl_3$) of Koninginin A (KA)

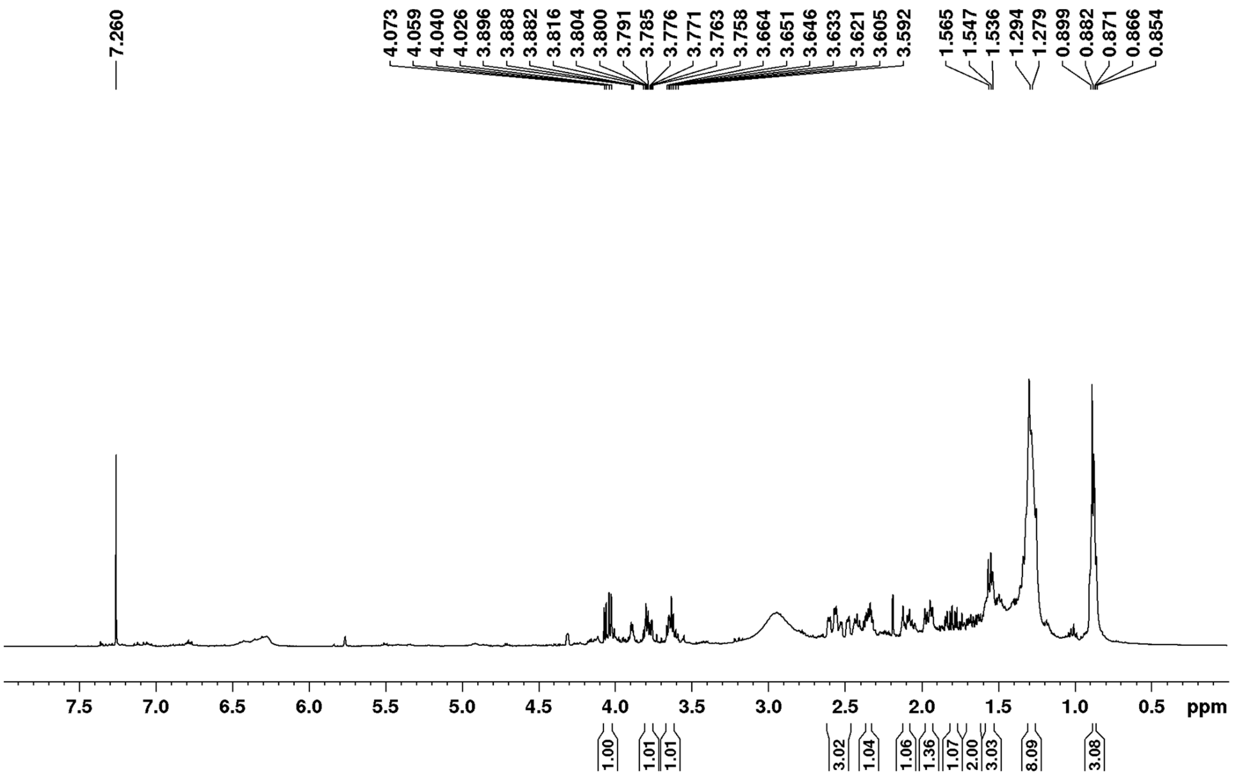

Figure S3: ^{1}H NMR spectrum (400 MHz, $CDCl_3$) of Koninginin B (KB)

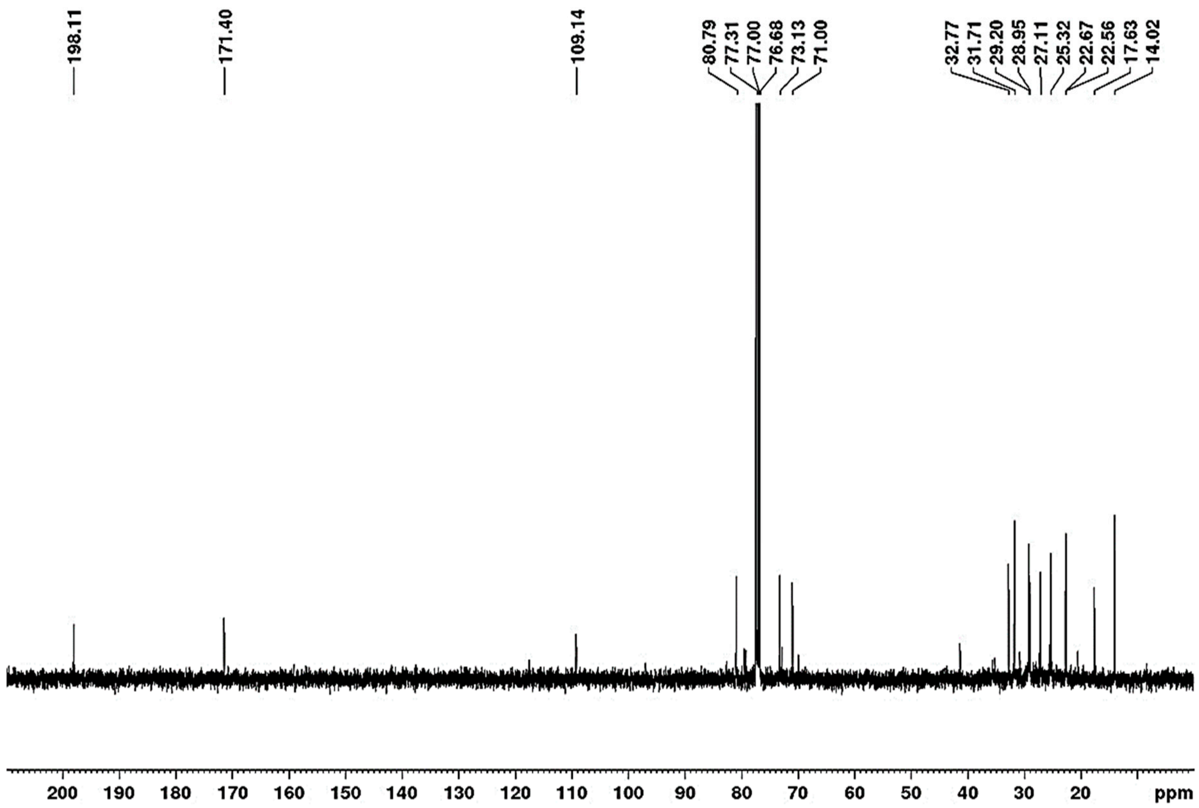

Figure S4: ^{13}C NMR spectrum (100 MHz, $CDCl_3$) of Koninginin B (KB)

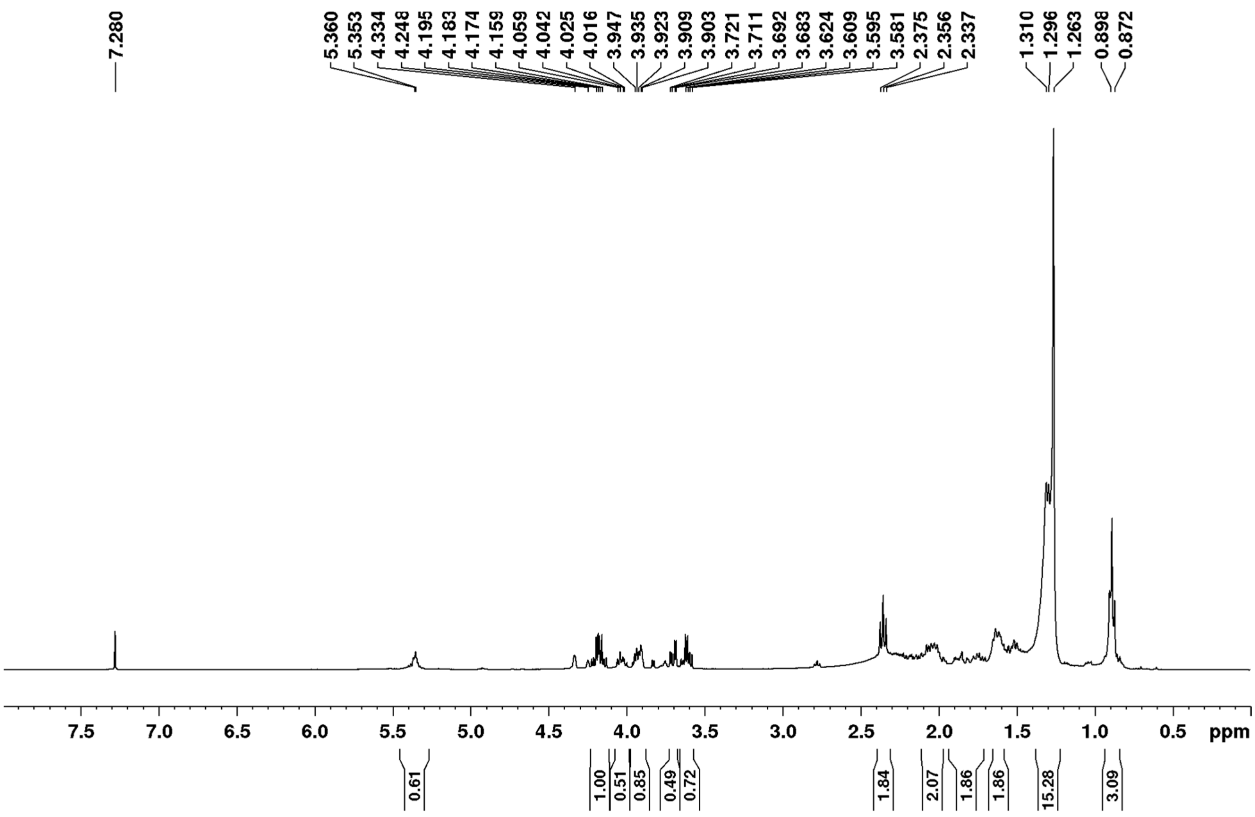

Figure S5: ^{1}H NMR spectrum (400 MHz, $CDCl_3$) of Koninginin C (KC)

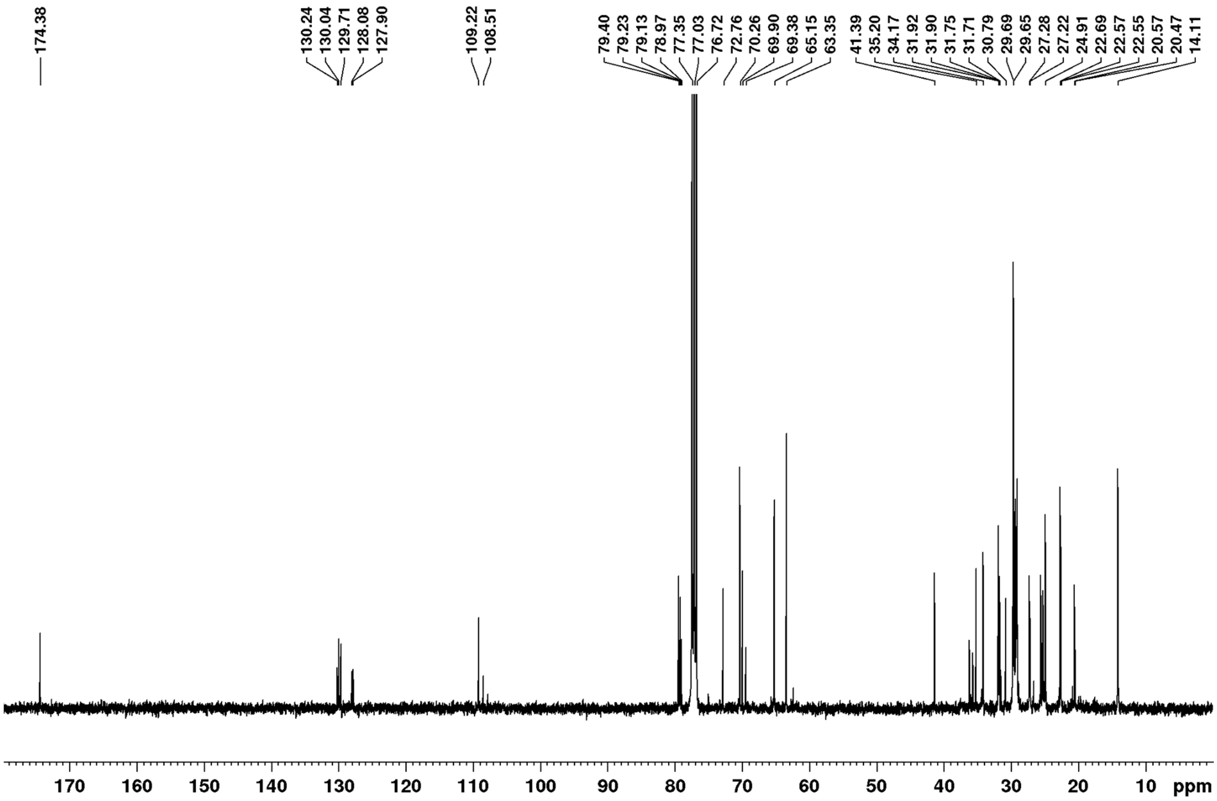

Figure S6: ^{13}C NMR spectrum (100 MHz, $CDCl_3$) of Koninginin C (KC)

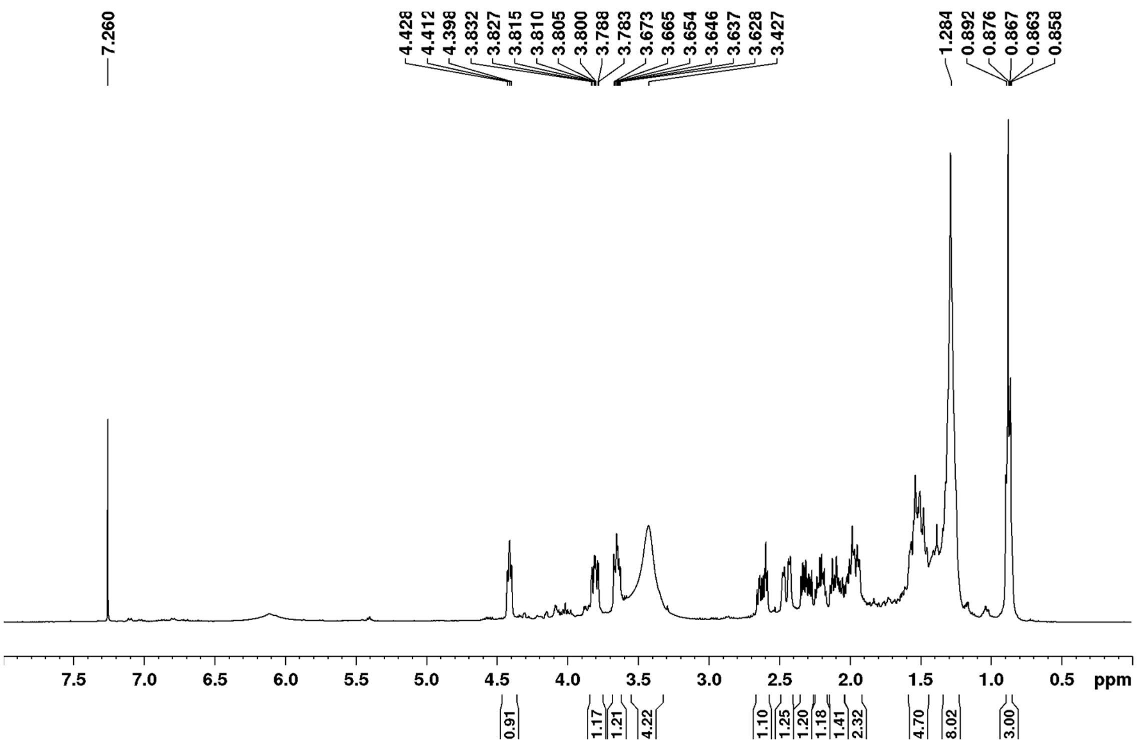

Figure S7: ^{1}H NMR spectrum (400 MHz, $CDCl_3$) of Koninginin E (KE)

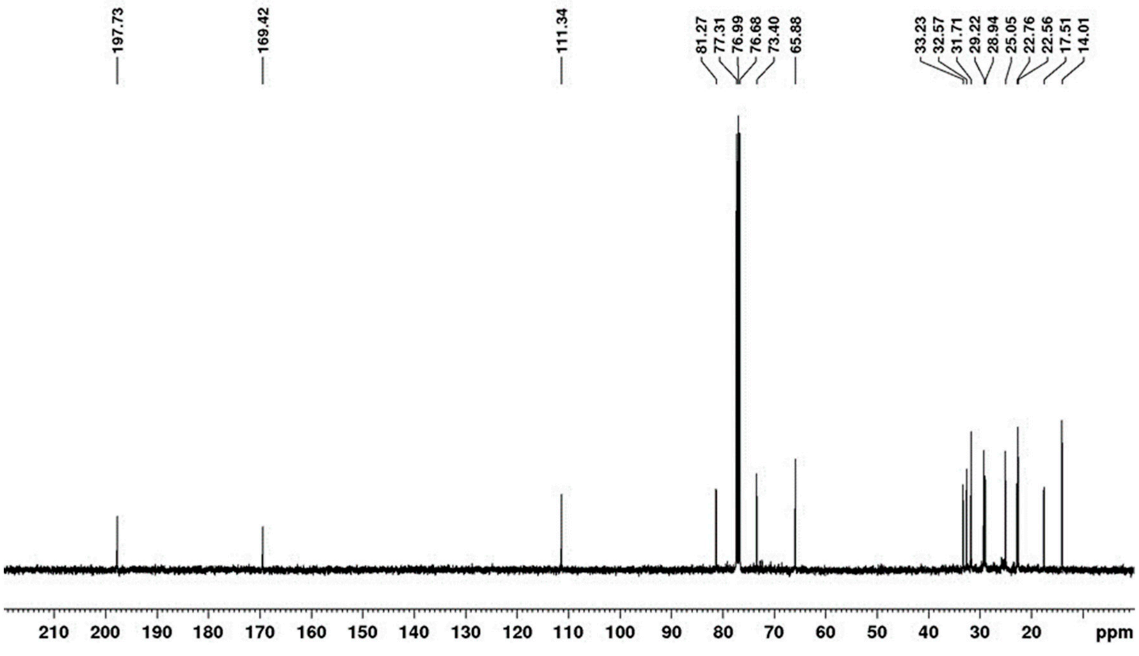

Figure S8: ^{13}C NMR spectrum (100 MHz, $CDCl_3$) of Koninginin E (KE)

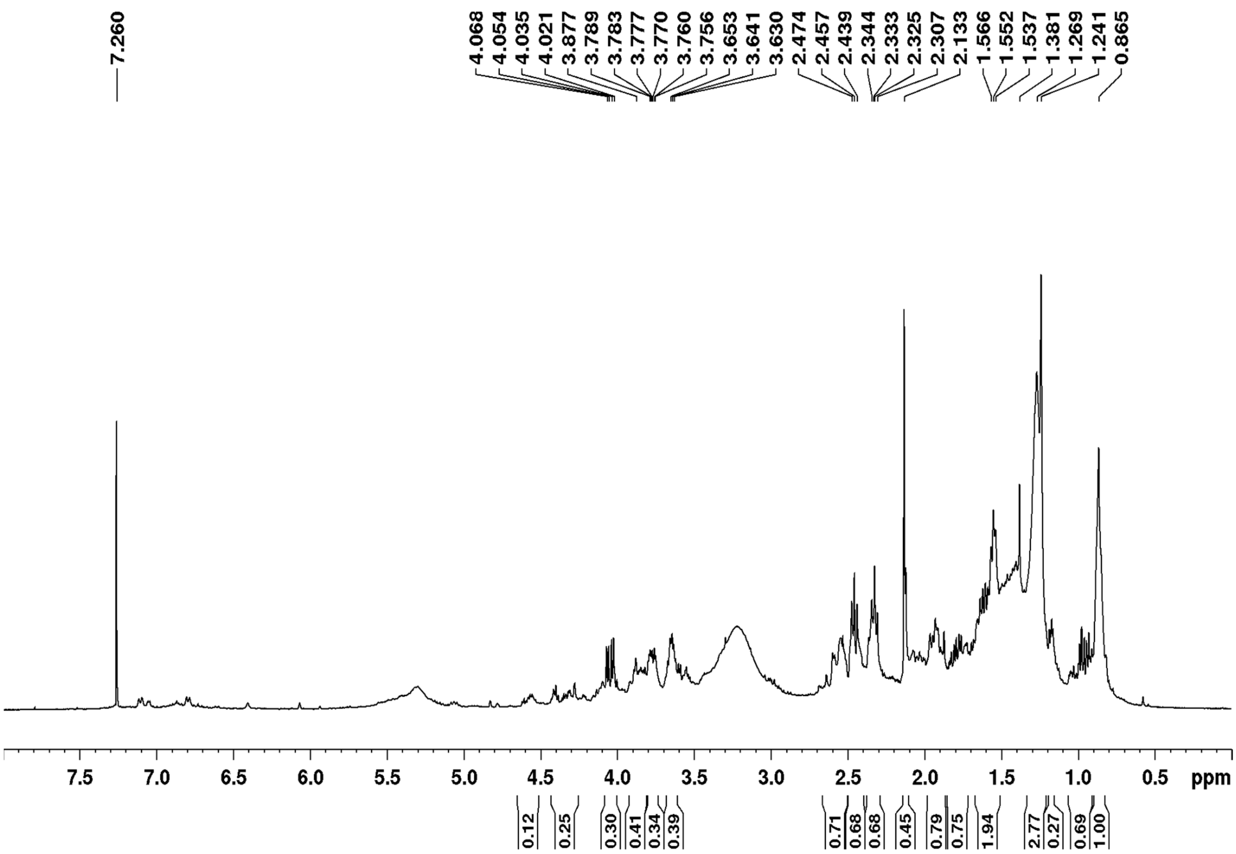

Figure S9: ^{1}H NMR spectrum (400 MHz, $CDCl_3$) of Koninginin J (KJ)

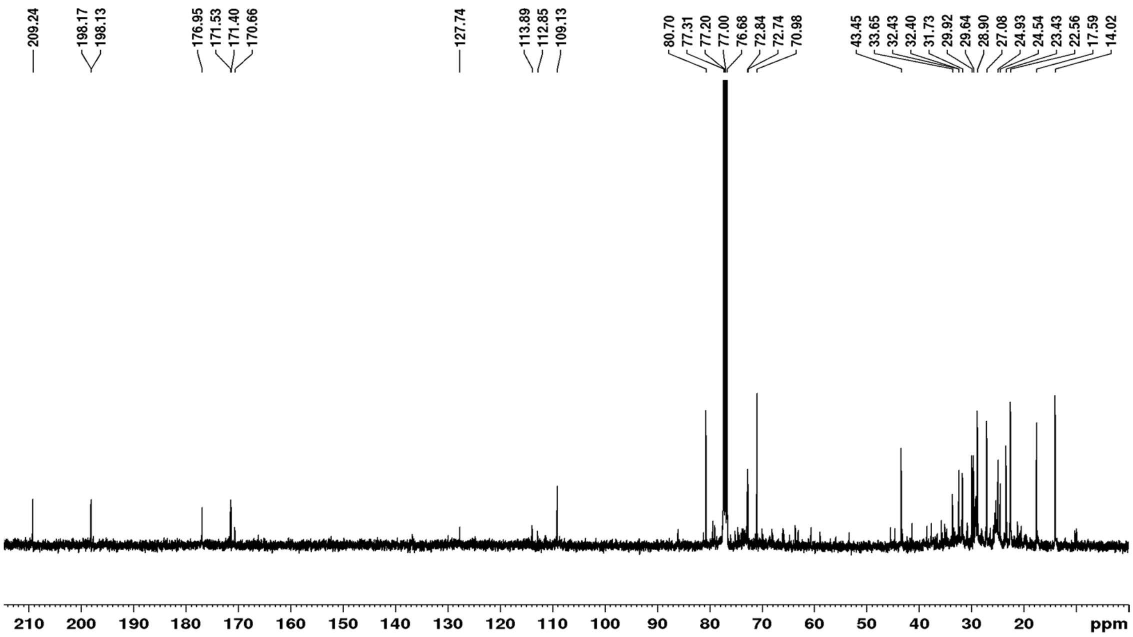

Figure S10: ^{13}C NMR spectrum (100 MHz, $CDCl_3$) of Koninginin J (KJ)

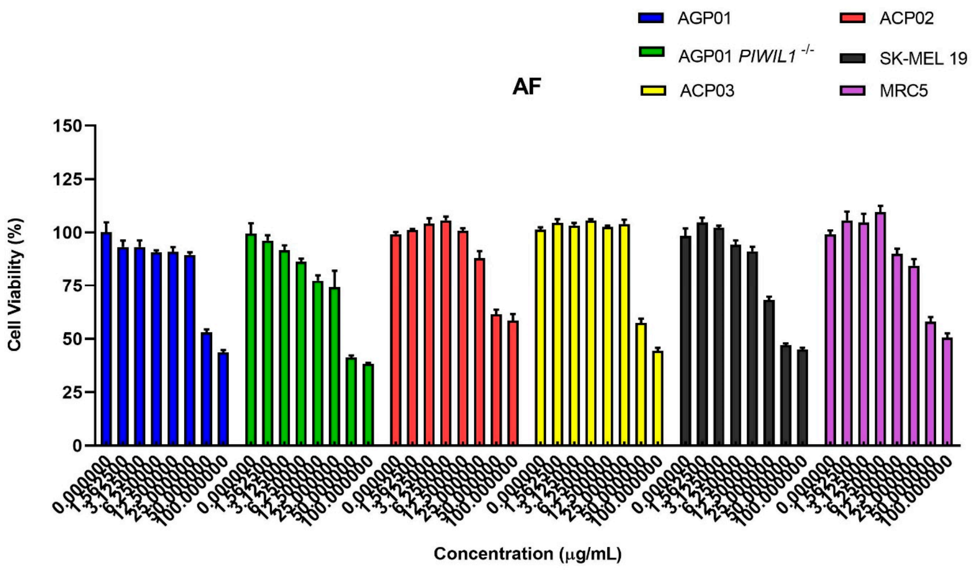

Figure S11. Cell viability percentage curve after 72 hours of treatment with AF extract in different cell lines*. Each point equals the average of three replicates.

*AGP-01 (intestinal-type gastric adenocarcinoma), AGP-01 PIWIL1 -/- (intestinal-type gastric adenocarcinoma with inactivated *PIWIL1* gene)1, ACP02 (diffuse-type gastric adenocarcinoma) and ACP03 (diffuse-type gastric adenocarcinoma), SK -MEL19 (Human Metastatic Melanoma), MRC5 (Normal Human Fibroblasts).

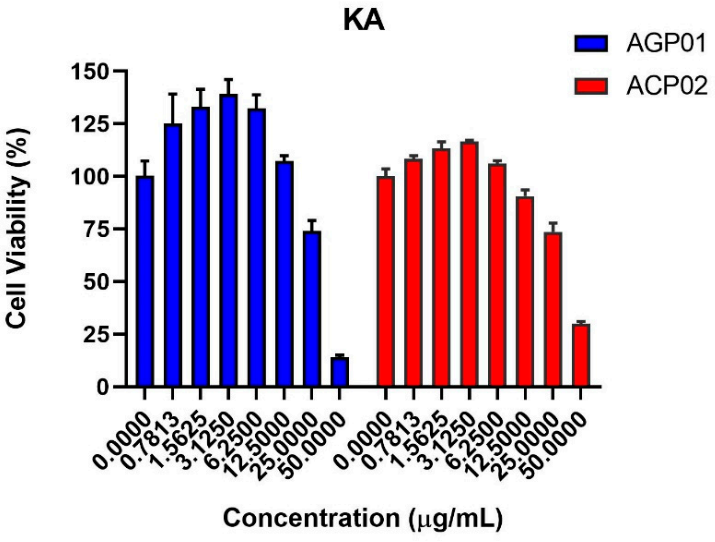

Figure S12. Cell viability percentage curve after 72 hours of treatment with koninginin A (KA) in two gastric cancer cell lines*. Each point equals the average of three replicates.

*AGP-01 (intestinal-type gastric adenocarcinoma), ACP02 (diffuse-type gastric adenocarcinoma).
